# Efficacy of knotless barbed versus conventional sutures for uterine closure during cesarean section: A prospective comparative study

**DOI:** 10.6026/973206300214929

**Published:** 2025-12-15

**Authors:** Akanksha Lamba Thora, Ankita Pawar, Anupama Dave

**Affiliations:** 1 Department of Obstetrics and Gynaecology, MTH Hospital, Indore Madhya Pradesh, India; 2 Department of Obstetrics and Gynaecology, MTH Women's Hospital, MGM Medical College, Indore, Madhya Pradesh, India

**Keywords:** Cesarean section, Conventional suturing, knotless barbed sutures

## Abstract

Cesarean section is a typical surgical practice whereby a good uterine closure is essential for maternal outcomes. Conventional suturing involves knot-tying, which may take long and may weaken the integrity of tissues. This study involves a comparison between knotless barbed sutures (KBS) and the use of conventional sutures (CS) to close the uterus in case of cesarean delivery. In an 80-women prospective comparative trial, KBS decreased the uterine incision closure time (8.58 ± 1.18 min vs. 10.18 ± 1.81 min, p=0.002) and total closure time (27.77 ± 4.73 min vs. 30.95 ± 3.08 min, p=0.001) significantly. The extra hemostatic sutures required were significantly reduced in KBS (5% vs. 27.5, p=0.006). The thickness of the scar at 6 weeks (4.23 ± 0.90 mm vs. 3.54 ± 0.60 mm, p<0.001), was larger in the KBS. Knotless barbed sutures have considerable benefits on efficiency of operations, hemostasis and tissue healing of cesarean uterine closure.

## Background:

Cesarean delivery (CD) is also one of the most commonly conducted major surgical operations in the world with the rates continuously rising [1]. Optimal uterine closure is of utmost importance in reducing short term complications like hemorrhage and long term integrity of the uterine scar that affects subsequent pregnancies [2, 3]. Conventional methods of uterine closure by suturing usually entail continuous or discontinuous sutures that necessitate the knot weaving. Although efficient, knot-tying is not only time-consuming, but also technically challenging in the context of minimal invasiveness, and can also cause potential risks, including knot slipping, excessive tension resulting in ischemia of the tissues, or localized hypoxia that will impair the healing of the wounds [4, 5]. Knotless barbed sutures (KBS) have been invented as a major breakthrough in wound closure technology with bidirectional barbs running through their length. These sutures were approved by the US Food and Drug Administration (FDA) in 2004 and implanted into tissue without need of knots to evenly distribute the tension along the suture line [6, 7]. The design has potential benefits such as decreased closure duration; fewer knot complications, and possible enhanced approval and healing of tissue [8, 9]. Although KBS have received a lot of acceptance and have been proven to be beneficial in laparoscopic and gynecological surgery, such as myomectomy and hysterectomy, their use in the open surgeries, especially in the use of uterine closure during cesarean section, is not well researched [10, 11]. Recent articles show that KBS has potential success in obstetric settings. Research has shown decreases in operating time and similar or even better results of blood loss and wound healing with the application of KBS in uterine closure over conventional sutures (CS) [12, 13]. Nevertheless, the FDA questions the efficacy and safety of KBS when used as a fascial closure, and there is limited strong data on large randomized trials in open cesarean surgery, which demonstrate the high gap in research work [14, 15]. Moreover, the influence of KBS on uterine scar integrity in the long-term is another important parameter that should be clarified by objective methods such as sonographic evaluation of scar thickness. Therefore, it is of interest to compare the two methods regarding the uterine incision closure time, additional hemostatic sutures used and approximated loss of blood.

## Materials and Methods:

## Study design and setting:

A prospective, comparative study was conducted over one year at the Department of Obstetrics and Gynaecology, MGM Medical College and MTH Hospital, following approval from the institutional ethics committee.

## Sample size calculation:

The sample size was calculated to detect a significant difference of 100 mL in the mean estimated blood loss (EBL) between groups, assuming a standard deviation (SD) of 156 mL. Using the formula for two independent means (α = 0.05, power = 80%, Zα/2 = 1.96, Zβ = 0.84), the required sample size per group was 40. To account for potential dropouts, a total sample size of 80 patients was targeted.

## Participants and selection criteria:

## Inclusion criteria: 

Pregnant women scheduled for caesarean delivery via Pfannenstiel incision meeting the following criteria: Primigravida or multipara with previous normal vaginal deliveries only; Full-term singleton pregnancies (≥37 weeks); Indications for CD including cephalopelvic disproportion, malpresentation, prelabor rupture of membranes (PROM), prolonged pregnancy, or fetal distress; Provision of written informed consent.

## Exclusion criteria: 

Refusal to provide consent; Suspected placenta previa or abruptio placentae; Known anterior lower uterine segment myomas; Multiple pregnancies; History of prior classical or inverted T caesarean incision; Maternal use of drugs affecting coagulation; Patients undergoing general anesthesia; Patients requesting concurrent tubal ligation; Presence of diabetes, fever, active systemic infection, or preeclampsia/eclampsia at the time of surgery; Body Mass Index (BMI) > 42 kg/m^2^; History of keloid or hypertrophic scar formation; Dermatological conditions known to impair wound healing; Use of immunosuppressive drugs; Chronic alcohol or drug abuse.

## Randomization and Intervention:

Eligible consenting patients were randomized into two groups (1:1 ratio) using the odd-even method based on hospital registration number:

[1] Group 1 (KBS): Uterine closure performed using a standardized knotless barbed suture (e.g., Stratafix™, Ethicon or V-Loc™, Medtronic).

[2] Group 2 (CS): Uterine closure performed using conventional polyglactin 910 (Vicryl™, Ethicon) suture.

All surgeries were performed under spinal anesthesia by experienced obstetricians adhering to a standardized modified Misgav-Ladach technique. The uterine incision was closed in two continuous layers for both groups. The first layer approximated the deep myometrium and decidua, and the second layer approximated the superficial myometrium and serosa. Care was taken to ensure precise tissue handling and approximation in both groups.

## Outcome measures:

## Primary outcomes:

[1] Uterine Incision Closure Time: Time (minutes) from the start of the first uterine suture placement to the completion of the second layer, measured using a stopwatch.

[2] Need for Additional Hemostatic Sutures: Requirement for any extra interrupted sutures placed specifically to control bleeding from the uterine incision edges or suture line after the initial continuous closure.

[3] Estimated Blood Loss (EBL): Calculated by measuring the volume in the suction canister and subtracting the volume of amniotic fluid and irrigation fluid, plus the estimated blood loss from soaked laparotomy pads (each fully soaked pad estimated as 100 mL, half-soaked as 50 mL).

## Secondary outcomes:

[1] Total Closure Time: Time (minutes) from skin incision to completion of skin closure.

[2] Postoperative Morbidity: Incidence of endometritis (defined as fever ≥38°C on two occasions >24 hours apart, uterine tenderness, foul lochia, and/or leukocytosis), wound infection, intestinal obstruction/volvulus, re-operation for hemorrhage, or readmission within 6 weeks.

[3] Uterine Scar Healing: Assessed via transvaginal sonography at 6 and 12 weeks postpartum. The primary sonographic parameter was residual myometrial thickness (RMT) at the thinnest point of the scar, measured in millimeters (mm) by a blinded sonographer using a standardized protocol.

## Data collection and follow-up:

Preoperative data (demographics, obstetric history, BMI, gestational age) were recorded. Intraoperative data (closure times, EBL, need for additional sutures) were collected prospectively. Patients were followed up at 6 and 12 weeks postpartum for clinical assessment of wound healing and transvaginal sonography.

## Statistical analysis:

Data were analyzed using SPSS version 23.0. Continuous variables (age, BMI, gestational age, closure times, EBL, scar thickness) were presented as mean ± standard deviation (SD). Categorical variables (parity distribution, need for additional sutures, morbidity) were presented as frequencies and percentages. Normality was assessed using Shapiro-Wilk test. Independent samples t-test was used to compare continuous variables between groups. Chi-square (χ^2^) test or Fisher's exact test (where expected frequencies <5) was used to compare categorical variables. A p-value < 0.05 was considered statistically significant. Risk Ratios (RR) with 95% Confidence Intervals (CI) were calculated for key binary outcomes.

## Results:

A total of 80 women undergoing caesarean section were enrolled and randomized: 40 to the KBS group and 40 to the CS group. All participants completed the study protocol and were included in the analysis. The groups were comparable regarding maternal age, BMI, and parity distribution. However, the mean gestational age was significantly lower in the KBS group compared to the CS group (p=0.016). Uterine incision closure time and total closure time were significantly shorter in the KBS group. The need for additional hemostatic sutures was significantly lower in the KBS group (RR=0.18, 95% CI 0.04-0.79). Although the mean EBL was lower in the KBS group, the difference did not reach statistical significance (p=0.067). Residual myometrial thickness at the uterine scar site was significantly greater in the KBS group compared to the CS group at both 6 weeks and 12 weeks postpartum (p<0.001 for both) (Table 1 - see PDF and 2 - see PDF). No cases of endometritis, wound infection, intestinal obstruction/volvulus, or need for re-operation for hemorrhage was observed in either group during the 6-week follow-up period (Table 3 - see PDF).

## Discussion:

This prospective comparative research has presented a strong evidence of the tremendous benefit of knotless barbed sutures (KBS) over traditional sutures (CS) in the segmentation of the uterus during a caesarean delivery. The greatest results were the significant decreases in uterine incision close-up period and the total surgical close-up time with KBS. The mean time of uterine closure was found to be about 1.6 minutes shorter (8.58 min vs. 10.18 min) and the overall time of uterine closure was more than 3 minutes shorter (27.77 min vs. 30.95 min) with the KBS group. This efficiency improvement is in line with the principle mechanism of KBS: the process of knot-tying is removed and this is known to be a time-consuming process in suturing [16, 17]. Other studies have also reported similar changes in the time spent in closing during the use of KBS [12, 13 and 18]. There is a positive correlation between the shorter the closure time and the shorter the anesthesia time and the general cost of surgery, which increases the efficiency of the operating room. One of the most important findings was that the extra hemostatic sutures were much lower in the KBS group (5% and 27.5, p=0.006, RR=0.18). This is a strong indication of better intrinsic hemostatic control by superior means with the barbed suture technique. The two-way barbs are probably more stable, even tension spread along the total suture line, better approximating tissue edges, and pinching small bleeding vessels in a more effective manner than traditional sutures, which just depends on the safety of knots at the intervals [19]. Such minimization of the necessity of other interventions also helps save time and reduces tissue manipulation, which can create the risk of infection or the formation of a hematoma. Although the average amount of blood that was estimated to have been lost was less in the KBS group (601 mL vs. 632 mL), the difference was not significant (p=0.067). This observation is in line with the results of some past studies [20, 21] but opposite to others where there were substantial changes in EBL as a result of KBS [12, 22]. This relatively small difference could be due to effectiveness in standard surgical hemostatic measures used in the two groups or the sample size being insufficient to measure relatively small but possibly clinically important difference in EBL.

The conclusion of long term uterine scar integrity test through transvaginal sonography demonstrated a very high importance to KBS. Myometrial residual thickness was much larger in the KBS group at 6 weeks (4.23 mm vs. 3.54 mm) and 12 weeks (4.73 mm vs. 3.97 mm) after childbirth. Thicker residual myometrium is commonly considered to be an important predictor of superior scar healing and strength, which is associated with a decreased occurrence of complications during later pregnancies, including uterine rupture or scar dehiscence [23, 24]. Increased scar thickness using KBS may be due to a greater uniform tension of the tissues and better anchoring offered by the barbs, which encourages ideal tissue apposition and possibly eliminates local inflammation or ischemia as compared to the focal pressure points made by knots using CS [25, 26]. This result helps to address an important issue of caesarean outcomes and allows the long-term safety profile of KBS in the context of uterine closure. Interestingly, there was not even a single postoperative morbidity (endometritis, infection, re-operation) in each group, as indicated by other studies, where KBS was not identified as worse than CS in terms of causing infectious and other comorbidities [27, 28]. Age, BMI and parity were well-matched baseline characteristics between groups and enhanced the validity of the outcome comparisons. The statistically significant difference in gestational age (KBS: 38.4 weeks, CS: 39.2 weeks, p=0.016) although statistically significant is a clinically insignificant difference (less than one week) within the term range and would not have confounded the main results involving suturing technique and closure efficiency. The nulliparous women in both groups were also high (60-67.5%), which was in line with the study population profile.

## Conclusion:

This prospective comparative study demonstrates that KBS significantly reduce uterine incision closure time, total operative closure time, and the need for additional hemostatic sutures compared to CS during caesarean delivery. Furthermore, KBS yield superior uterine scar healing, evidenced by greater residual myometrial thickness at 6 weeks and 12 weeks, with no postoperative complications in either group. These findings support KBS as a safe, efficient alternative to CS for uterine closure, potentially optimizing maternal outcomes and warranting larger randomized trials for broader adoption.
